# Evaluating Clinical Outcomes in Children With Acute Osteomyelitis Treated With Intravenous Versus Oral Antimicrobial Therapy

**DOI:** 10.7759/cureus.104551

**Published:** 2026-03-02

**Authors:** Tiffany Withrow, Hannah Lu, Ankita Desai, LeAnne Tripp

**Affiliations:** 1 Department of Pharmacy Services, Children's Medical Center Dallas, Dallas, USA; 2 Department of Pediatric Pharmacy, University Hospitals Rainbow Babies and Children's Hospital, Cleveland, USA; 3 Department of Pediatric Infectious Diseases, University Hospitals Rainbow Babies and Children's Hospital, Cleveland, USA

**Keywords:** antibiotics, bone and joint infections, children, infectious diseases, osteomyelitis, pediatrics

## Abstract

Introduction and aim: Osteomyelitis is an infection of the bone that typically requires hospitalization for diagnosis and initial treatment. For many years, it has been assumed that osteomyelitis must be treated with intravenous (IV) antibiotics for the full course of therapy. However, recent data have demonstrated the efficacy of oral (PO) antibiotics. In addition, studies comparing the efficacy of IV versus PO antibiotics in children with osteomyelitis have found that there was no increased incidence of treatment failure when patients were transitioned to PO antibiotics. The Infectious Diseases Society of America 2021 guideline on the management of acute hematogenous osteomyelitis recommends transitioning from IV to PO regimens when an acceptable PO antibiotic is available. This study aimed to evaluate the efficacy and use of IV versus PO antibiotic therapy for the treatment of osteomyelitis at a pediatric tertiary care center.

Methods: This retrospective chart review evaluated patients under 18 years of age admitted from January 2019 to December 2021 with a diagnosis of acute osteomyelitis. Patients were grouped according to the route of antimicrobial administration prescribed at hospital discharge.

Results: Fifty-one patients with osteomyelitis were included, with 19 and 32 patients in the PO and IV groups, respectively. There was no difference in the treatment failure rate between the two groups (IV=3 {9.4%} versus PO=2 {10.5%}; p=1.000). Patients in the IV group had higher rates of adverse drug events compared to the PO group (IV=9 {28.1%} versus PO=4 {21.1%}), with diarrhea and drug-induced neutropenia being the most common.

Conclusion: Oral therapy for osteomyelitis may be associated with fewer adverse events than IV therapy, with no difference in treatment failure.

## Introduction

Osteomyelitis is an infection of the bone that usually requires hospitalization for diagnosis and initial treatment [1]. The incidence of acute hematogenous osteomyelitis (AHO) in children is variable and ranges from 1.2 to 13 cases per 100,000 children per year [2]. In children, osteomyelitis typically involves a single bone and most commonly occurs in the long bones of the arms and legs [2]. In North America, *Staphylococcus aureus (S. aureus)* is the most common pathogen causing AHO in both children and adults [2]. For many years, it has been assumed that osteomyelitis must be treated with intravenous (IV) antibiotics for the full course of therapy [3]. However, this is based on limited data, and recent literature has demonstrated the efficacy of oral (PO) antibiotics [3,4]. Adult patients with osteomyelitis are still more commonly treated with IV antibiotics. In children with osteomyelitis, studies comparing the efficacy of IV versus PO antibiotics have found that there is no increased incidence of treatment failure when patients were transitioned to PO antibiotics [5-9]. The 2021 guideline published by the Infectious Diseases Society of America (IDSA) on the management of AHO in children recommends transitioning from IV to PO regimens when an acceptable PO antibiotic is available [2].

This study aimed to compare the clinical outcomes and adverse effects associated with IV versus PO antibiotic therapy at discharge in children with acute osteomyelitis at a pediatric tertiary care center. This study was part of a broad quality improvement initiative at the institution to decrease the use of indwelling catheters and home IV therapy for pediatric patients. Studies suggest that IV therapy is associated with more adverse events compared to PO therapy [5,8,10]. The objective of this study was to compare the treatment failure rates between IV and PO therapy. Treatment failure was defined as the occurrence of any of the following within six months of completion of antimicrobial therapy for osteomyelitis: revisit to the emergency department (ED) related to osteomyelitis, rehospitalization for a change in the antibiotic prescribed or its dosage, prolongation of antibiotic therapy, conversion from the PO to the IV route, need for bone abscess drainage after initial treatment period, need for debridement of necrotic bone, bone biopsy, drainage of an abscess of the skin or muscle after initial treatment period, arthrocentesis, or diagnosis of a pathologic fracture after initial treatment period. Secondary objectives included adverse drug events, peripherally inserted central catheter (PICC)-related complications, and total duration of antibiotic use. Given the lack of clear guidelines on when to transition to oral therapy, this study sought to evaluate outcomes of real-world prescribing practices to inform institutional protocols and reduce unnecessary IV therapy.

## Materials and methods

Population

This was a retrospective chart review conducted at Rainbow Babies and Children’s Hospital and was approved by the University Hospitals Institutional Review Board. Patients under 18 years of age admitted between January 2019 and December 2021 were included if they had a diagnosis code of acute osteomyelitis. International Classification of Diseases, Tenth Revision (ICD-10) codes were obtained from the electronic health record billing department at the institution and included the following: M86.0 (AHO), M86.1 (other acute osteomyelitis), and M86.2 (subacute osteomyelitis). Patients were classified into either the IV or PO group based on the route of antimicrobial therapy prescribed at discharge. The following patients were excluded: immunocompromised patients requiring treatment with IV antibiotics for the duration of treatment, treatment for an infection with IV antibiotics within the past six months, osteomyelitis due to non-bacterial pathogens (example: fungus), patients with osteomyelitis secondary to a sacral decubitus ulcer, and patients with sickle cell disease. In addition, patients were excluded if they received their entire antibiotic regimen inpatient, or if they received IV antibiotics for indications other than osteomyelitis during their initial therapy or within the six months following initial therapy.

Data collection

Data included patient age at admission, sex, site of infection, surgical interventions, cultures, radiographic results, antibiotic regimen, hospital length of stay, duration of PICC placement, duration of antibiotic therapy, and reported adverse events related to the antibiotic or PICC.

Statistical analysis

Statistical analysis was performed using SPSS version 25.0 (Armonk, NY: IBM Corp.) software. Numerical data were compared for median equality via Wilcoxon-Mann-Whitney U tests. Categorical data were compared for distributional equality via Fisher's exact tests except for gender, which employed Pearson’s chi-square test. Statistical significance was set at p<0.05.

## Results

**Figure 1:**
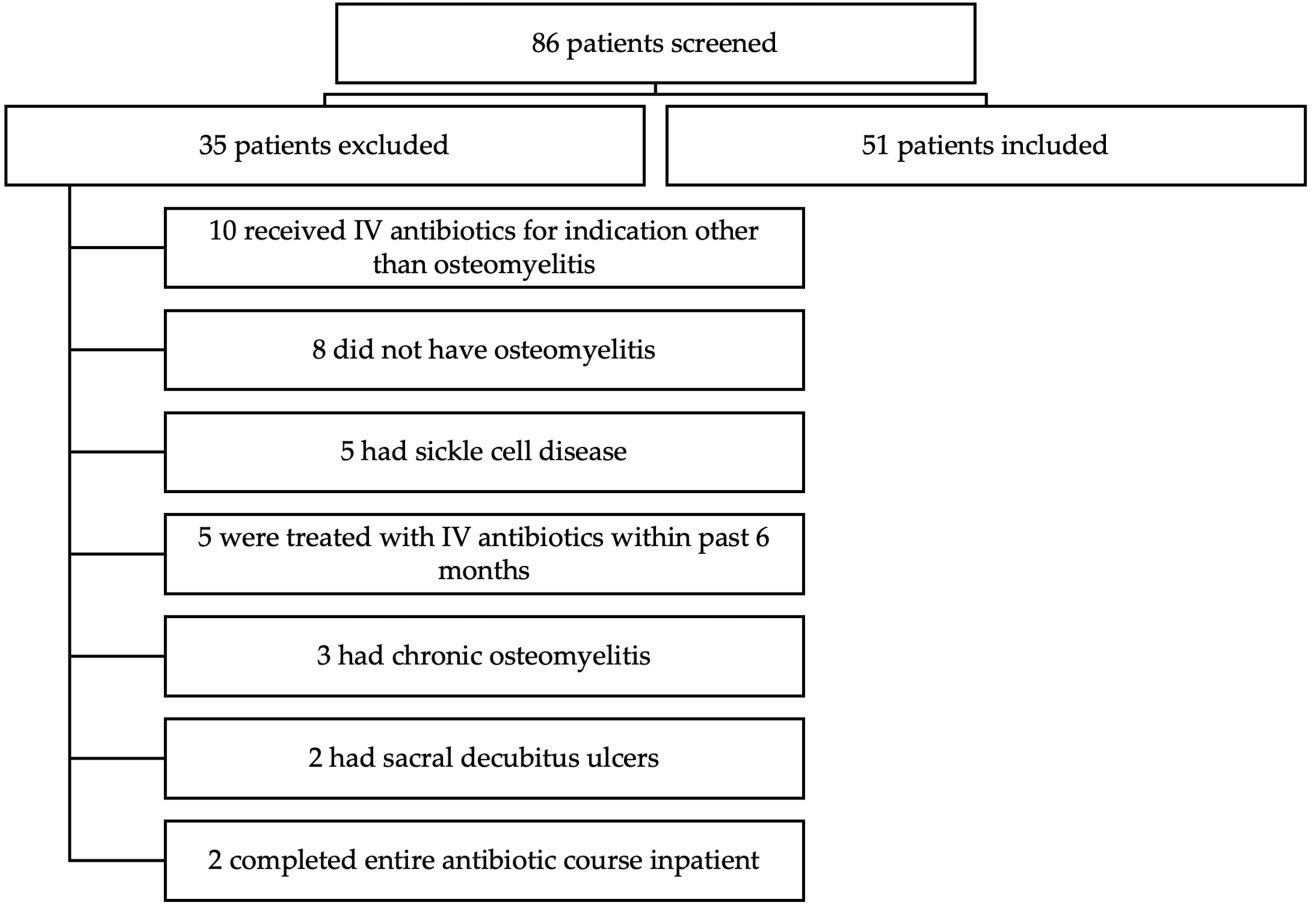
Study inclusion flowchart.

A total of 86 patients had a diagnosis code of acute osteomyelitis during the study period and were screened for study inclusion. As shown in Figure 1, 51 patients were included in the final analysis. Baseline demographics are shown in Table 1. Thirty-two patients were discharged on IV antibiotics, and 19 patients were discharged on PO antibiotics. The median age was eight years in the IV group and 11 years in the PO group. Of note, there was one neonate and one infant in the IV group and one infant in the PO group. Two patients (3.9%) had a documented drug allergy to an antimicrobial agent; one patient in each group. The patient in the PO group had an anaphylactic reaction to azithromycin, and the patient in the IV group had an allergy to sulfonamides documented due to glucose-6-phosphate dehydrogenase deficiency. Sites of osteomyelitis infection for each group are shown in Table 1. The most common sites of infection for both groups were the lower extremities (IV=20 {62.5%} versus PO=15 {78.9%}).

**Table 1:** Baseline demographics and site of osteomyelitis infection.

Variables	IV (n=32)	PO (n=19)	p-Value
Age (years), median (IQR)	8 (3-12)	11 (5-13)	0.446
Male, n (%)	21 (65.6)	15 (78.9)	0.313 (chi-square=1.109, df=1)
Documented antimicrobial drug allergy, n (%)	1 (3.1)	1 (5.3)	-
Patient with a positive blood culture, n (%)	17 (53.1)	0	<0.001
Site of osteomyelitis infection, n (%)	-	-	0.058
1. Lower extremity (leg/hip/foot/knee/ankle)	20 (62.5)	15 (78.9)	-
a. Leg	11 (34.4)	6 (31.6)	-
b. Hip	5 (15.6)	1 (5.3)	-
c. Leg/hip	0	1 (5.3)	-
d. Leg/knee	0	1 (5.3)	-
e. Foot	3 (9.4)	5 (26.3)	-
f. Ankle	1 (3.1)	1 (5.3)	-
2. Upper extremity (arm/elbow/hand)	2 (6.3)	3 (15.8)	-
a. Arm	1 (3.1)	1 (5.3)	-
b. Elbow	1 (3.1)	0	-
c. Hand	0	2 (10.5)	-
3. Spine	6 (18.8)	0	-
4. Other/not specified	4 (12.5)	1 (5.3)	-

**Figure 2:**
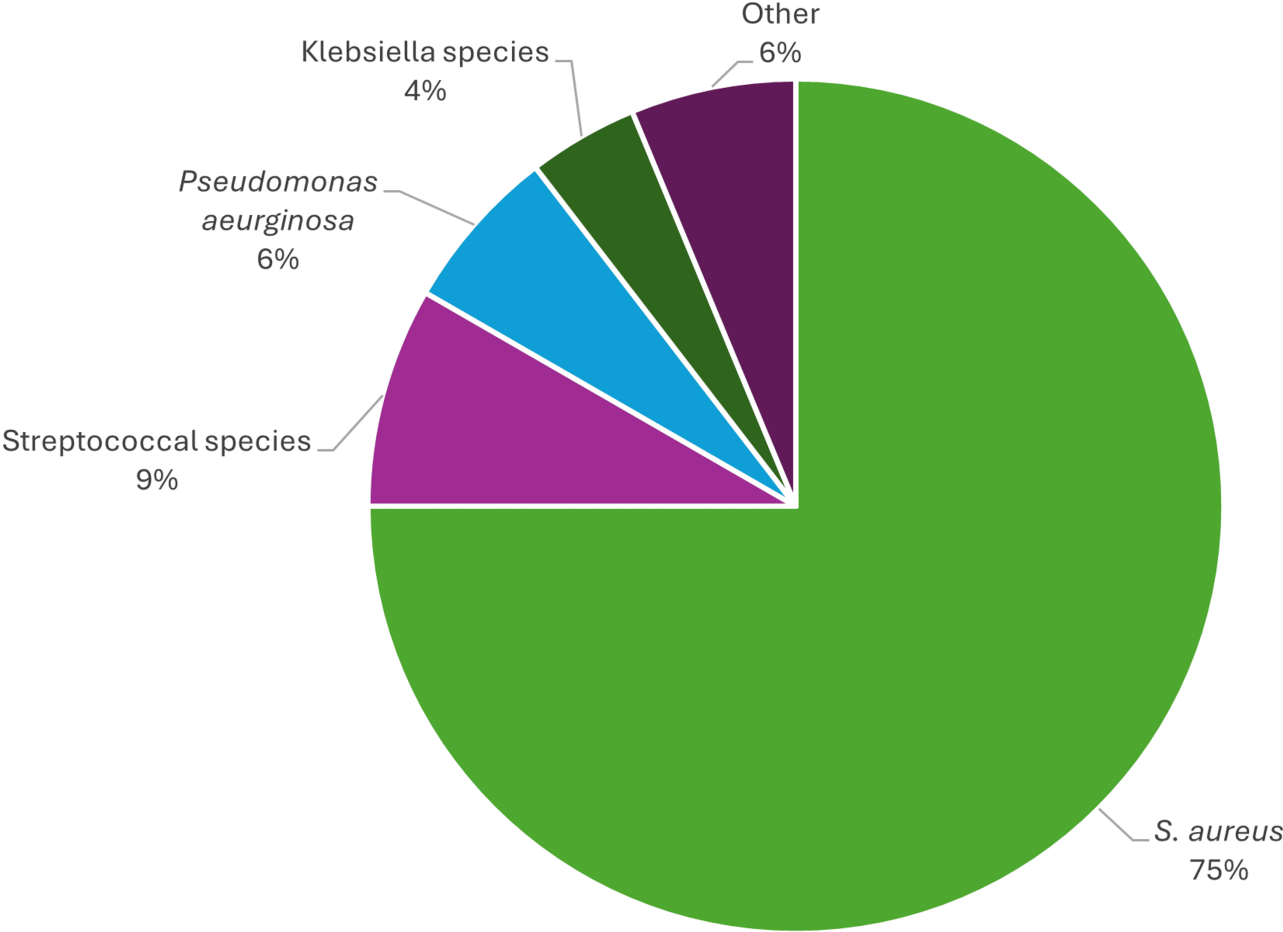
Isolated pathogens from all cultures.

There was a total of 34 patients in the study population with a positive culture - 27 in the IV group and seven in the PO group. As shown in Table 1, 53.1% of the included patients in the IV group had positive blood cultures; however, none of the patients in the PO group had a positive blood culture (p<0.001). Table 2 depicts the cultures and microbiology data. In the IV group, 17 patients (53.1%) had positive blood cultures, 19 patients (59.4%) had positive bone and wound cultures, and nine patients (28.1%) had both. Seven patients (36.8%) in the PO group had positive bone and wound cultures. Table 2 illustrates that *S. aureus* was the most common pathogen for both the IV and PO groups for bone and wound cultures (n=14 {43.8%} and 7 {36.8%}, respectively). Among patients in the IV group with positive blood cultures, *S. aureus *was also the most common pathogen identified (n=15; 88.2%). Of the individuals with positive blood cultures growing *S. aureus*, methicillin-susceptible *S. aureus* (MSSA) was more prevalent than methicillin-resistant *S. aureus* (MRSA) (n=12 {80%} versus n=3 {20%}, respectively). Figure 2 displays the distribution of bacterial pathogens from all cultures, highlighting that *S. aureus *accounted for the largest proportion of infections.

**Table 2:** Summary of microbiological data. *There were nine patients with concomitant bacteremia and a positive bone or wound culture. MSSA: methicillin-susceptible *S. aureus*; MRSA: methicillin-resistant *S. aureus*

Variables	IV (n=32)	PO (n=19)
Number of positive blood cultures, n (%)	17 (53.1)	0
1. *S. aureus*, n (%)	15 (46.9)	-
a. Methicillin-susceptible (MSSA), n (%)	12 (37.5)	-
b. Methicillin-resistant (MRSA), n (%)	3 (9.4)	-
2. Streptococcal species, n (%)	1 (3.1)	-
3. *Klebsiella aerogenes*, n (%)	1 (3.1)	-
Number of positive bone and wound cultures, n (%)	19 (59.4)	7 (36.8)
1. Positive blood cultures, n (%)*	9 (28.1)	0
2. Polymicrobial, n (%)	3 (9.4)	1 (5.3)
3. *S. aureus*, n (%)	14 (43.8)	7 (36.8)
a. MSSA, n (%)	9 (28.1)	5 (26.3)
b. MRSA, n (%)	5 (15.6)	2 (10.5)
4. *Pseudomonas aeruginosa*, n (%)	3 (9.4)	0
5. Streptococcal species, n (%)	2 (6.3)	1 (5.3)
6. *Escherichia coli*, n (%)	1 (3.1)	0
7. *Klebsiella oxytoca*, n (%)	1 (3.1)	0
8. *Enterobacter cloacae*, n (%)	1 (3.1)	0
9. *Aeromonas hydrophila*, n (%)	1 (3.1)	0

For the primary outcome of treatment failure, a total of five patients had treatment failure - three (9.4%) patients in the IV group and two (10.5%) patients in the PO group (p=1.000) (Table 3). The median number of days from hospital discharge to treatment failure was longer in the PO group than in the IV group (88 versus eight days, respectively). Secondary outcomes, as shown in Table 3, include the incidence of adverse effects and total duration of antibiotic therapy. The median duration of antibiotics was longer in the IV group than that of the PO group (47.5 days versus 43 days). Patients in the PO group received a median of three days of inpatient IV therapy before transitioning to PO therapy. Each patient in the IV group had a PICC placed, of which seven (21.9%) had a PICC-related complication. The PICC-related complications included two deep vein thromboses, two dislodgements, line occlusion, pain, bleeding, and irritation. The median duration of PICC placement was 17.5 days. There were three patients in the PO group who had a PICC placed, all of whom were placed during the patient’s initial hospitalization and were removed prior to hospital discharge. The median duration of days for PICC placement for these patients was five, and there were no documented PICC-related complications. There were nine patients (28.1%) in the IV group and four patients (21.1%) in the PO group who experienced an adverse drug event. The most common adverse drug events were diarrhea, drug-induced neutropenia, and leukopenia (Table 3).

**Table 3:** Primary and secondary outcomes. *One patient in the IV group experienced acute kidney injury, fever, and transaminitis. One patient in the IV group experienced drug-induced neutropenia and leukopenia. One patient in the PO group experienced diarrhea and nausea. PICC: peripherally inserted central catheter

Variables	IV (n=32)	PO (n=19)	p-Value
Treatment failure, n (%)	3 (9.4)	2 (10.5)	1.0
95% CI (%)	0-19.9	0-24.3	-
Days from hospital discharge to treatment failure (median)	8	88	-
Revisit to the emergency department related to osteomyelitis	2 (6.3)	0	-
Readmission for a change in antibiotic therapy	1 (3.1)	0	-
Prolongation of antibiotic therapy	0	2 (10.5)	-
Overall duration of antibiotics (days), median (IQR)	47.5 (44-55)	43 (33-88)	-
1. Total IV therapy (days), median (IQR)	20 (16-35)	3 (2-5.25)	-
a. Inpatient IV therapy (days), median (IQR)	6 (5-8.75)	3 (2-5.25)	-
b. Outpatient IV therapy (days), median (IQR)	14 (10-24)	0 (0-0)	-
2. Total PO therapy (days), median (IQR)	29.5 (26.5-34.75)	39 (29-82)	-
a. Inpatient PO therapy (days), median (IQR)	0 (0-0)	0.5 (0-1)	-
b. Outpatient PO therapy (days), median (IQR)	29.5 (26.5-34.75)	39.5 (29.75-83.25)	-
Placement of PICC, n (%)	32 (100)	3 (15.8)	-
1. Duration (days), median (IQR)	17.5 (11.5-29.5)	5	-
2. Complication, n (%)	7 (21.9)	0	-
Adverse drug event, n (%)*	9 (28.1)	4 (21.1)	-
1. Diarrhea	3 (9.4)	3 (15.8)	-
2. Drug-induced neutropenia	3 (9.4)	1 (5.3)	-
3. Leukopenia	2 (6.3)	-	-
4. Nausea	-	1 (5.3)	-
5. Vomiting	1 (3.1)	-	-
6. Allergic reaction	1 (3.1)	-	-
7. Acute kidney injury	1 (3.1)	-	-
8. Fever	1 (3.1)	-	-
9. Transaminitis	1 (3.1)	-	-
10. Skin peeling	1 (3.1)	-	-

First-generation cephalosporins were the most common antimicrobial prescribed at discharge in both the IV and PO groups (Table 4). For the IV group, cefazolin was the most common (n=15 {46.9%}), followed by ceftriaxone (n=6 {18.8%}) and clindamycin (n=3 {9.4%}). For the PO group, cephalexin was the most common (n=10 {52.6%}), followed by clindamycin (n=3 {15.8%}) and ciprofloxacin (n=3 {15.8%}).

**Table 4:** Antimicrobials at discharge.

Variables	IV (n=32)	PO (n=19)
Amoxicillin/clavulanate	-	1 (5.3)
Azithromycin	-	1 (5.3)
Cefazolin	15 (46.9)	-
Cefepime	2 (6.3)	-
Ceftazidime	1 (3.1)	-
Ceftriaxone	6 (18.8)	-
Cephalexin	-	10 (52.6)
Ciprofloxacin	-	3 (15.8)
Clindamycin	3 (9.4)	3 (15.8)
Daptomycin	1 (3.1)	-
Doxycycline	-	1 (5.3)
Ertapenem	1 (3.1)	-
Linezolid	1 (3.1)	1 (5.3)
Metronidazole	1 (3.1)	-
Piperacillin/tazobactam	1 (3.1)	-

## Discussion

Although multiple studies have demonstrated the efficacy of PO antimicrobials for pediatric osteomyelitis [5-7,11], guidelines have yet to provide clear recommendations on when to transition a patient from IV to PO therapy [2]. In this study, patients were assessed based on the route of antimicrobials prescribed at the time of their hospital discharge. Among those with a positive culture for bacterial etiology of osteomyelitis, the majority were positive for *S. aureus.* First-generation cephalosporins were the most commonly prescribed antimicrobials at discharge for both the IV and PO groups. For the primary outcome, there was no difference in the incidence of treatment failure between the two groups. This confirms findings from previous studies assessing treatment failures for those treated with PO antibiotic therapy. A study in Finland by Pääkkönen et al. included 265 pediatric patients with culture-proven acute bone or joint infection who were initially treated with IV antimicrobials and then switched to PO therapy within four days [6]. All patients in this study were transitioned to PO therapy with clindamycin, amoxicillin, or a first-generation cephalosporin [6]. Although there was no comparison group, this study demonstrated the successful treatment of pediatric patients with bone or joint infections with PO therapy after two to four days of IV therapy [6]. A retrospective study conducted by Kargel et al. evaluated 21 children with osteomyelitis of the hand treated exclusively with PO antimicrobials, which included cephalexin, clindamycin, sulfamethoxazole-trimethoprim, amoxicillin-clavulanate, and ciprofloxacin [12]. Culture positivity was 38% in the children in the study, with* S. aureus *being the most common pathogen [12]. The study concluded that most cases of osteomyelitis of the hand can be successfully treated with PO antimicrobials [12]. The PO antimicrobials from both of these studies were similar to the PO antimicrobials prescribed in our study, with the majority of our patients being discharged on cephalexin, ciprofloxacin, or clindamycin (Table 4).

A study by Keren et al. of 2060 pediatric patients demonstrated that children who were treated with PO therapy did not experience more treatment failures than those treated with IV therapy (5% versus 6%, respectively) (p=0.77) [5]. There was a statistically significantly higher overall incidence of treatment-related adverse events in the IV group compared to that of the PO group (p<0.001), which was largely due to PICC-related complications [5]. This resulted in an increase in visits to the ED and rehospitalization due to an adverse effect in the IV group as compared to the PO group (risk difference, 14.6%, 95% CI: 11.3-17.9%) [5]. In a multicenter, open-label, parallel-group, randomized controlled trial conducted by Li et al., switching to PO therapy after seven days of IV therapy to complete a total of six weeks of antimicrobial therapy was non-inferior to IV therapy in adults with osteomyelitis [8]. Additionally, the study demonstrated IV antibiotics were associated with increased hospital length of stay, PICC-related complications, and earlier discontinuation of treatment [8]. A study by Ruebner et al. found that 41% of pediatric patients with osteomyelitis treated with IV therapy for more than two weeks experienced a line-related complication [13]. Another study by Murphy et al. concluded that IV agents were associated with a four-fold increased incidence of adverse events and a five-fold increase when including venous access catheters [14]. In our study, patients in the IV group had a higher incidence of adverse drug events compared to the PO group (IV=9 {28.1%} and PO=4 {21.1%}). Furthermore, 21.9% of patients in the IV group had a PICC-related complication. These findings are consistent with other studies that found higher rates of complications and adverse events with prolonged use of IV therapy when compared with PO therapy [5,8,10,14].

The results of our study support the use of early transition to PO antimicrobial therapy for the treatment of pediatric osteomyelitis. McNeil et al. examined the treatment practices and outcomes of *S. aureus *bacteremic osteoarticular infections in 192 children and concluded that orthopedic complications were not increased for those transitioned to PO antibiotics [9]. This study also noted that patients with positive blood cultures were less often transitioned to PO therapy than patients without positive blood cultures. Our study had similar results, as all of the patients with bacteremic infections were prescribed IV therapy at discharge. A retrospective cohort study across multiple pediatric hospitals evaluated opportunities to switch from IV to PO antibiotics [15]. An opportunity to switch was defined as a day in which a patient received both an IV and PO antibiotic [15]. The results of this study indicated that there was an opportunity to switch to PO antibiotics on more than half of the days a patient was receiving antibiotics [15]. While this study included several types of infection, the percent opportunity (defined as the aggregate percent of days patients received IV or PO antibiotics that were opportunity days) was 69% for osteomyelitis and 73% for septic arthritis [15]. A 2023 analysis by Mehler et al. concluded that the average duration of IV treatment was reduced following the implementation of a pediatric infectious diseases (ID) consultation service [16]. Each of the patients in our study had the ID service consulted. While we did not collect data on how often ID recommended transitioning to PO therapy, this would have been interesting to assess. These studies, paired with our results, highlight the need for more guidance for providers to transition to oral therapy.

It is well-known that exposure to antimicrobials can have adverse effects, including increased risk of atopic dermatitis, asthma, juvenile idiopathic arthritis, psoriasis, and neurocognitive disorders [17]. Additionally, a study by Ramirez et al. details the negative effects that antibiotics can have on gut microbiota, including reduced species diversity and selection for antibiotic-resistant organisms [18]. Diarrhea was one of the most common adverse effects for both of our groups, with an incidence of 9.4% for the IV group and 15.3% for the PO group. Authors Solis and Dehority reported that neutropenia was common among children receiving antimicrobial therapy for osteoarticular infections, with an incidence of 32.8% [19]. While the incidence of neutropenia in our study was much lower (9.4% in the IV group and 5.3% in the PO group), it was still one of the most common adverse events. A 2021 retrospective observational study by Same et al. determined that more than one in five antimicrobial courses were complicated by adverse events [20]. This is similar to the incidence in our study, with an incidence of 28.1% in the IV group and 21.1% in the PO group. The total duration of antibiotic therapy was longer in the IV group than in the PO group; however, both groups had an average duration longer than six weeks. A systematic review by Howard-Jones and Isaacs suggested that a total duration of three weeks may be as effective as longer courses for patients with uncomplicated acute osteomyelitis [21]. A six-year retrospective study by Filleron et al. also had success with shorter antimicrobial courses [22]. The authors of that study successfully treated 151 pediatric patients with osteomyelitis and septic arthritis over 14 days [22]. The authors reported no treatment failures; however, 7% of patients required secondary surgical revision, which would have been classified as a treatment failure under the criteria for our study [22]. A shorter duration of therapy may reduce the incidence of adverse effects and complications associated with antimicrobial therapy. While our study was not designed to assess the duration of antimicrobial therapy or its impact on adverse effects, this would be an important direction for future studies as the transition to PO therapy increases.

Notably, only one individual (2.0%) had a documented anaphylactic reaction to an antimicrobial, which is lower than the reported prevalence of penicillin allergy alone, which ranges from 5% to 10% [23]. No patients had a documented anaphylactic allergy to a beta-lactam antibiotic, including amoxicillin, which is reportedly the most common drug allergy in children [23]. To our knowledge, this study is the only one to have assessed the status of antimicrobial allergy in pediatric osteomyelitis. Limitations of this study include a small sample size, a retrospective study design, and an analysis limited to a single center. We had to rely on the diagnosis codes and the accuracy of the information documented in the electronic health record; therefore, there may have been additional adverse effects or treatment failures that were not recorded by providers. This study also had a limited follow-up duration, ending six months after completion of antimicrobial therapy. All antimicrobials were assumed to be dosed appropriately, and adherence could not be assessed after patient discharge. Poor adherence or improper antimicrobial dosage may have impacted the study outcomes, including adverse effects and the incidence of treatment failure. Additionally, the small sample size of this study may limit its applicability in clinical practice.

## Conclusions

There was no difference in treatment failure rates between the IV and PO groups among pediatric patients with acute osteomyelitis. Patients in the IV group were more likely to experience an adverse drug event compared to patients in the PO group, with diarrhea and drug-induced neutropenia being the most common. Treatment of osteomyelitis with PO therapy may be associated with fewer adverse events than IV therapy, with no difference in rates of treatment failure. This study will be used as part of the institution’s quality improvement initiative to reduce the use of indwelling catheters and to increase the number of patients discharged on PO therapy.
